# Lipids alter microbial transport through intestinal mucus

**DOI:** 10.1371/journal.pone.0209151

**Published:** 2018-12-21

**Authors:** Taylor L. Carlson, Hasan Yildiz, Zaineb Dar, Jaclyn Y. Lock, Rebecca L. Carrier

**Affiliations:** 1 Department of Chemical Engineering, Northeastern University, Boston, Massachusetts, United States of America; 2 Antisense Oligonucleotide Manufacturing and Development, Biogen, Cambridge, Massachusetts, United States of America; 3 Department of Bioengineering, Northeastern University, Boston, Massachusetts, United States of America; "INSERM", FRANCE

## Abstract

Mucus constitutes a protective layer which coats the gastrointestinal tract, controlling interactions of both commensal and pathogenic microbes with underlying tissues. Changes to the mucus barrier, for example due to altered mucin expression or external stimuli, may impact interactions with microbes and thus potentially contribute to altered gut homeostasis, onset of inflammation, or pathogen invasion. Food-associated stimuli, including lipids, have been shown to change mucus barrier properties and reduce transport of model drug carriers through mucus. Here, we explore the impact of lipids, specifically triglycerides in a model intestinal medium mimicking a fed state, on *Escherichia coli* (*E*. *coli*) transport through mucus by directly imaging swimming patterns and analyzing associated changes in mucus structure. Lipids in model fed state intestinal contents reduced *E*. *coli* speed and track linearity within mucus. These changes may be due in part to changes in molecular interactions within the mucus network as well as crowding of the mucus network by lipid emulsion droplets, which visibly stay intact in the mucus gel. In addition, observed physical interactions between bacteria and lipid structures may impact microbial speed and trajectories. As lipids are normal food components and thus represent safe, mild stimuli, these results support exploration of lipid-based strategies to alter the mucus barrier to control interactions with microbes and potentially prevent microbial invasion of underlying epithelium.

## Introduction

Mucus lines the gastrointestinal tract (GIT) and acts as a physical barrier preventing microbial invasion of the underlying epithelium[1]. Mucins, antibodies (i.e. immunoglobulins), antimicrobial peptides, and lysozyme present in the complex, mesh-like mucus gel bind or kill microbes, allowing them to eventually be removed from the GIT as the mucus layer is cleared and replenished[2, 3]. Deficiencies or changes in the mucus layer can expose the epithelium, leading to microbial invasion and inflammation of underlying tissue[4, 5]. Food ingestion results in significant changes in the GIT lumen environment, impacting composition and transit rates of luminal material[6, 7], as well as mucus production[8, 9]. We have recently demonstrated that intestinal mucus barrier properties are significantly altered by model intestinal contents consisting of triglycerides, their digestion products, and bile components, representative of fed conditions in the GIT[10]. Exposure to lipids significantly hindered the transport of polymeric nanoparticles ranging in size from 20 to 1000 nm[11]. This result suggests alterations in the mucus barrier after oral consumption of lipids may aid in protecting the underlying epithelium from exposure to ingested particulates.

Given the important role of GIT mucus in controlling interactions between microbes and underlying epithelium, the focus of this study is to explore the impact of food-associated lipids on the transport of *Escherichia coli* (*E*. *coli*) through intestinal mucus. Trajectories of microbes in buffer and mucus in the presence and absence of lipids were tracked and quantitatively analyzed. A deeper understanding of mucus barrier alterations in the presence of food-associated lipids and other luminal stimuli could motivate development of treatments to modulate mucus and thus alter interactions with intestinal microbes, to minimize pathogen invasion and potentially mitigate gastrointestinal inflammation.

## Materials and methods

### Native intestinal mucus collection

Mucus was collected from porcine small intestine (Research 87 Inc., Boylston MA) within 2 hours of slaughter. After the intestine was rinsed with cold water, mucus was collected by gently scraping with a spatula and stored at -80°C for later use.

### Preparation and characterization of microbes and test media

*Escherichia coli* (*E*. *coli*) MG1655 was transformed *via* heat shock to express green fluorescent protein (GFP). Briefly, *E*. *coli* MG1655 from frozen stocks were cultured overnight in Luria-Bertani (LB) broth, pelleted (5000 rpm, 4 °C, 10 min.), re-suspended in calcium chloride (CaCl_2_, 100 mM, 30 min., 4 °C), and then re-pelleted and re-suspended in CaCl_2_ (2 ml). The GFP plasmid was isolated from *E*. *coli* DH5a using a QIAprepspin miniprep kit (Qiagen) and added to the *E*. *coli* solution before undergoing heat shock (42 °C, 45 sec.). Cells were plated on ampicillin positive LB plates, fluorescent colonies were collected and grown overnight in LB broth at 37 °C and 250 rpm, and then bacteria glycerol stocks (20% glycerol) were prepared and stored at -80 °C. GFP-expressing *E*. *coli* (GFP *E*. *coli*) (5 μl) from frozen stocks were grown overnight in LB (5 mL) at 37°C and 250 rpm prior to utilization in experiments as described below.

Model fed-state intestinal contents (referred to as “fed state” below, pH 6.5) were prepared with maleate buffer (MB) (pH 6.5), model bile, and lipids mimicking a partially digested triglyceride mixture (soybean oil, monoglyceride, and fatty acid) (Table 1). All chemicals were obtained from Sigma-Aldrich unless noted otherwise. Fed state media composition is designed to represent the physicochemical and physiological properties of GI tract contents [10, 12], and MB is the base buffer of this media, representing the absence of lipids and bile components characteristic of the fed state. The solution was mixed overnight at 37 °C with continuous magnetic stirring at 500 rpm. GFP *E*. *coli* (5 μl cells in LB, ~10^6^ cells/ml) were added to the MB or fed state (25 μl) and incubated for 30 min. at 37°C and 250 rpm. GFP *E*. *coli* in MB or fed state solution (7.5 μl) was dosed to MB or mucus (150 μl) in a well slide (FastWells, Grace Bio-Labs) to allow investigation of the impact of fed state medium on microbe mobility in the presence and absence of mucus.

**Table 1:** Fed state medium.

**Maleate Buffer**	Triz-Ma	100	mM
	NaCl	65	mM
	CaCl_2_	10	mM
	NaOH	40	mM
**Model Bile**	NaTDC	12	mM
	Lecithin	4	mM
**Lipid**	Soybean Oil	35	mM
	Sodium Oleate	30	mM
	1-Oleoyl-rac-glycerol	15	mM

### Video microscopy and bacteria tracking algorithm

**Fig 1:**
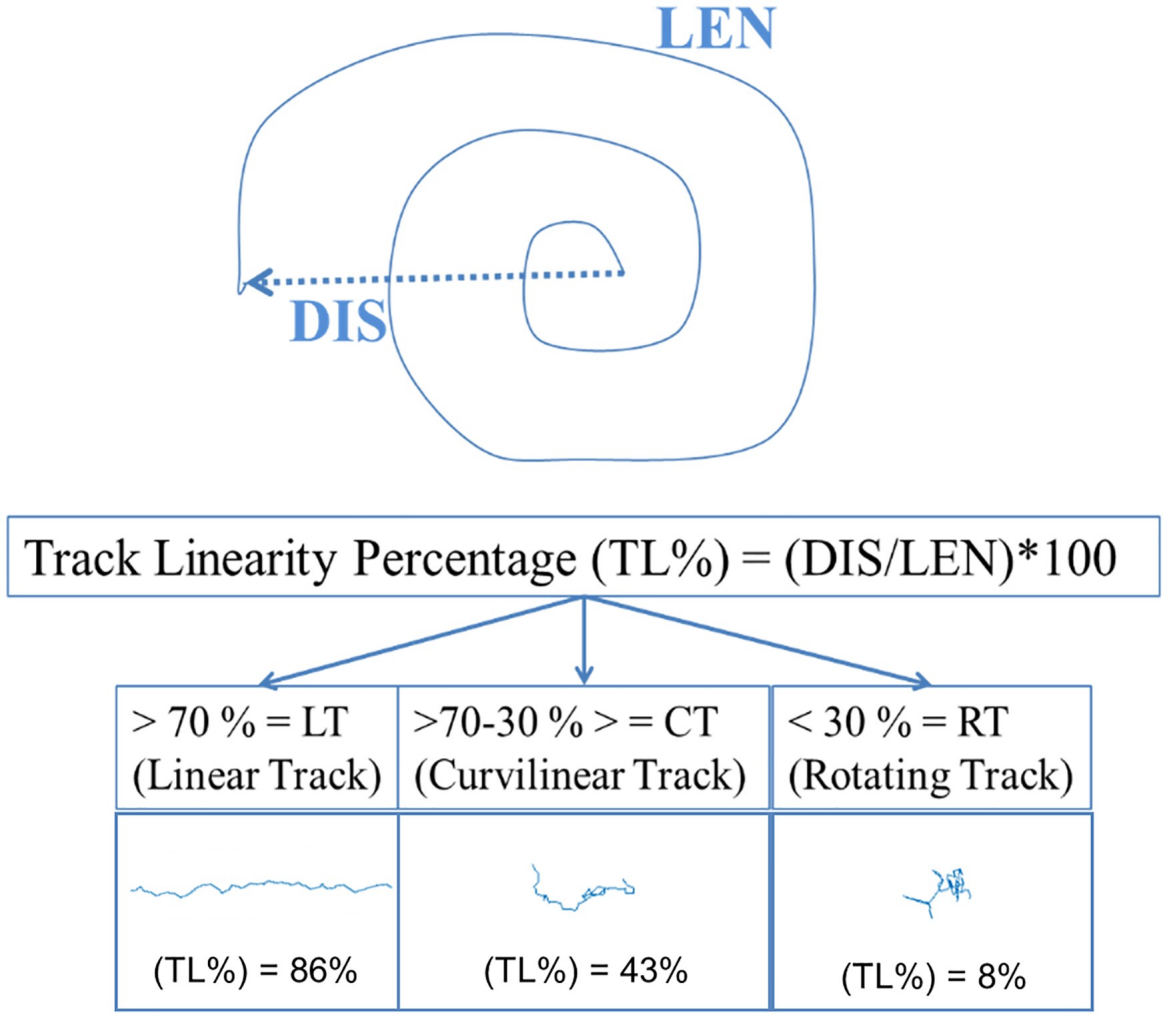
Classification of the linearity of bacterial trajectories. Figure was adapted from Pontier-Bres et al.[17].

Transport of individual bacteria was visualized using an Olympus DP70 digital color camera (Olympus) mounted on an inverted Olympus IX51 microscope with an attached X-Cite 120 fluorescence illumination system (EXFO). Bacteria transport videos were captured at 40X magnification with a frame rate of 30 fps for 20 s. A 220x206 pixel region of interest (ROI) selection was used to reduce the field of view and facilitate bacteria trajectory analysis. Individual bacterium trajectories were obtained using the ImageJ Mosaic plugin[13] and a modified version of the MATLAB tracking algorithm[14]. The algorithm was edited to account for elongated (rather than spherical) fluorescent entities since *E*. *coli* are rod-shaped[15]. Due to microscopy and tracking methodology limitations, tracked microbes may only stay in focus for a short period before disappearing from the region of interest. Only tracks which were captured for more than 50 frames and were not out of focus for more than 2 frames were analyzed for speed and movement characterization. Individual bacterium tracks were categorized based on track linearity (TL) which is defined as the ratio between the distance covered by a bacterium (DIS) and the length (LEN) of the bacterium track (Fig 1)[16]. A classification system developed by Pontier-Bres et al.[17] was used to categorize tracks as linear (LT) when the ratio was greater than 0.70, curvilinear (CT) when the ratio was between 0.30 and 0.70, and rotating (RT) when it was below 0.30.

Speeds of *E*. *coli* were calculated using Eq 1,

$$Speed=\frac{\sqrt{{\left[x(t+\tau )-x(t)\right]}^{2}+{\left[y(t+\tau )-y(t)\right]}^{2}}}{\tau }$$ (1)

where x and y represent microbe position at a given time (t) and τ is the time scale. Microbial speed distribution profiles were obtained and the overall average speeds were calculated over a time period of 1s. The total distance traveled at 1 s accounts for the distance traveled between each time interval (τ = 1/30 s).

### Imaging of lipid and microbe distribution

To allow visualization of relative positions of microbe trajectories and lipid droplets, lipid microemulsions in the fed state medium (25 μL), prepared as described above, were stained with BODIPY (TR Methyl Ester, 598/625 nm, 1 mg/mL, 0.5 μL, ThermoFisher Scientific) immediately before addition of GFP *E*. *coli* (5 μL cells in LB). After a 30 min. incubation, microbe transport was visualized in the fed state solution and in fed state solution (10 L) dosed to mucus (200 μL) by overlaying consecutively captured twenty-second videos obtained using FITC (475–490 nm) and TRITC (545–565 nm) filters. To minimize solution drift and aid in a clean overlay image, the fed state-GFP *E*. *coli* solution was placed between two coverslips for imaging without dilution in MB, which resulted in coalescing of lipids into larger emulsion droplets (up to approximately 60 μm) but facilitated visualization of bacterium interactions with droplet surfaces. *E*. *coli* interacted with the lipid droplets independent of size, but it was easier to observe these interactions (and they appeared to occur over longer periods of time) for droplets of larger size. In an effort to mimic conditions relevant to lipid-enriched mucus *in vivo*, microbe movement was quantified in diluted emulsions, as noted above.

For confocal imaging of lipid and microbe distribution within mucus, lipid microemulsions in the fed state medium (25 μL), prepared as described above, were stained with BODIPY (1 mg/mL, 0.5 μL) immediately before addition of GFP *E*. *coli* (5 μL cells in LB). GFP *E*. *coli* were incubated for 30 min in MB or in fed state solution at 37°C and 250 rpm, and then added (7.5 μL) to unstained mucus (150 μL) or mucus stained with 4 μL lectin [*Ulex europaeus*-FITC or *Ulex europaeus*-TRITC, 1 mg/mL]. These mucus samples were then incubated for 30 mins. and imaged with a Zeiss LSM 710 confocal microscope. Confocal z-stacks of the mucus layer were obtained at 20x, and ImageJ was used to generate maximum intensity z-stack projections.

### Mucus ultrastructure and pore size

Fed state medium (1 μL) was dosed to mucus (15 μL) in a well slide and incubated in a humid chamber for 2 hrs. The sample was then fixed by immersion in Carnoy’s fixative (60% ethanol, 30% chloroform, and 10% glacial acetic acid) for 1 hr and exchanged in 100% ethanol before being critical point dried and sputter coated. Scanning electron micrographs were taken using a Hitachi S-4800 scanning electron microscope with cold field emission gun, and ImageJ was then used to identify and quantify the ferret diameter of mucus pores.

### Lipid size characterization

Fed state medium was prepared as described above and diluted 1000x in MB. Samples were analyzed at 37°C with a N4 Plus Coulter counter from Beckman Coulter Inc., and the intensity-average diameters are reported.

### Viscosity

Fed state medium and MB were prepared as described above, and the solution viscosity was measured at 37 °C with a calibrated BS/IP/RF U-tube reverse flow viscometer (Fungilab, Part number: CV006-001).

### Statistics

Three separate experiments were performed, each with mucus from a different porcine intestine, with at least 100 microbes analyzed in each experiment per test medium. The average measurements from each individual experiment were obtained, and data are presented as the overall mean ± the standard error of the mean (SEM) of these average values obtained from separate experiments. Analysis of variance (ANOVA) and Student’s t-test were used to evaluate statistical significance. ANOVA was performed across all groups to assess statistical significance of impact of test media on microbe speed, percentages of linear, curvilinear, and rotating tracks.

## Results and discussion

To explore the impact of model fed state intestinal contents (“fed state” medium) on microbe penetration through gastrointestinal mucus, the impact of fed state medium on *E*. *coli* transport through mucus relative to impact on transport through buffer was studied. *E*. *coli* in either fed state medium or buffer (MB) were thus pipetted onto a mucus gel, or within MB for comparison, and *E*. *coli* mobility within the mucus gel or MB was analyzed utilizing fluorescence video microscopy.

### Impact of fed state medium on the transport of *E*. *coli* within MB

**Fig 2:**
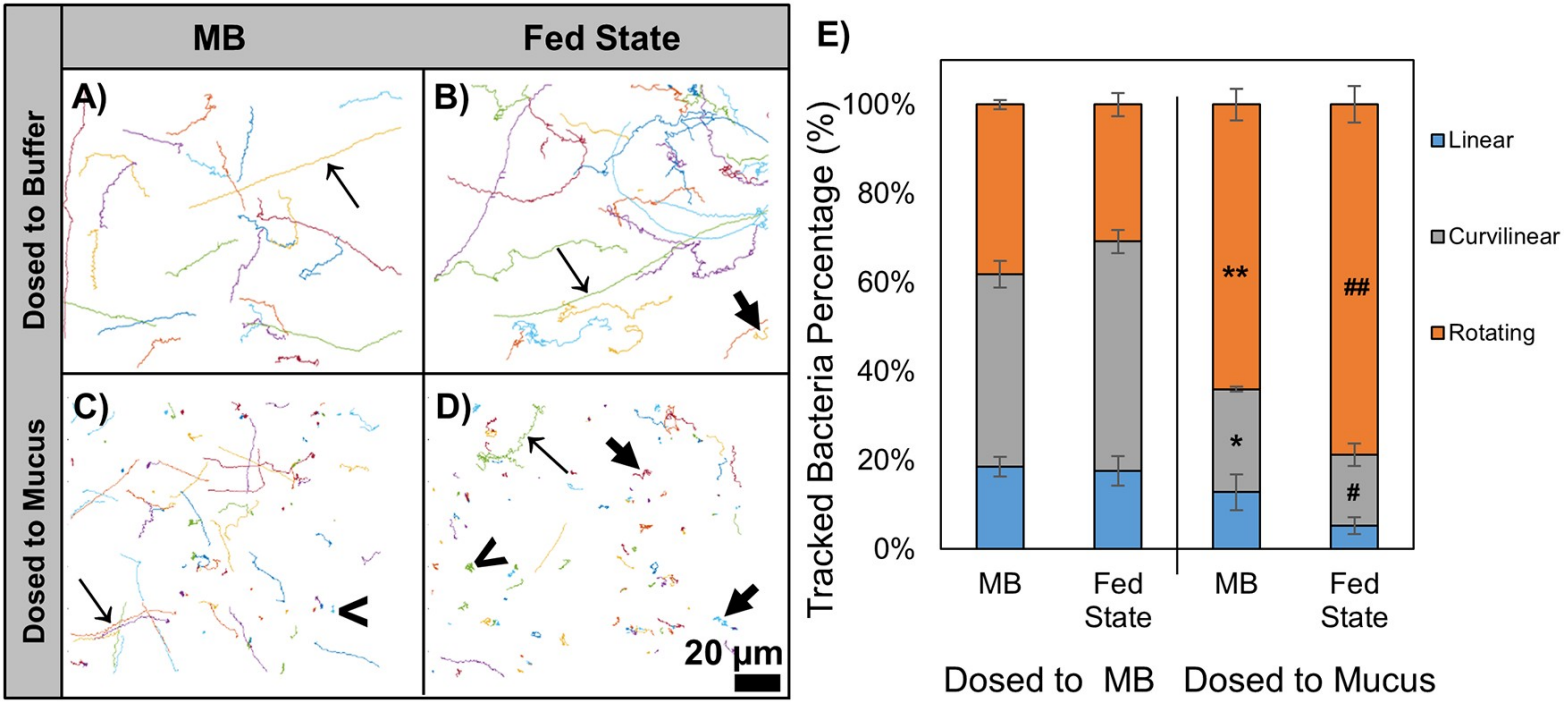
Representative 20 s trajectories of *E*. *coli* swimming in MB (A, B) or mucus (C, D) in the presence (B, D) or absence (A, C) of fed state medium. Linear (**→**), curvilinear (**➔**) and rotating (**<**) tracks are highlighted. Scale bar: 20 μm. E) Classification of bacteria movement by track linearity. When dosed to mucus (C, D), *E*. *coli* had a significant decrease in curvilinear tracks (* p<0.05) and increase in rotating tracks (** p<0.05) compared with dosage to MB (A, B). Fed state medium significantly increased rotating tracks (## p<0.05) and decreased curvilinear tracks (# p<0.05) in mucus (C, D). Scale bar = 20 μm.

**Fig 3:**
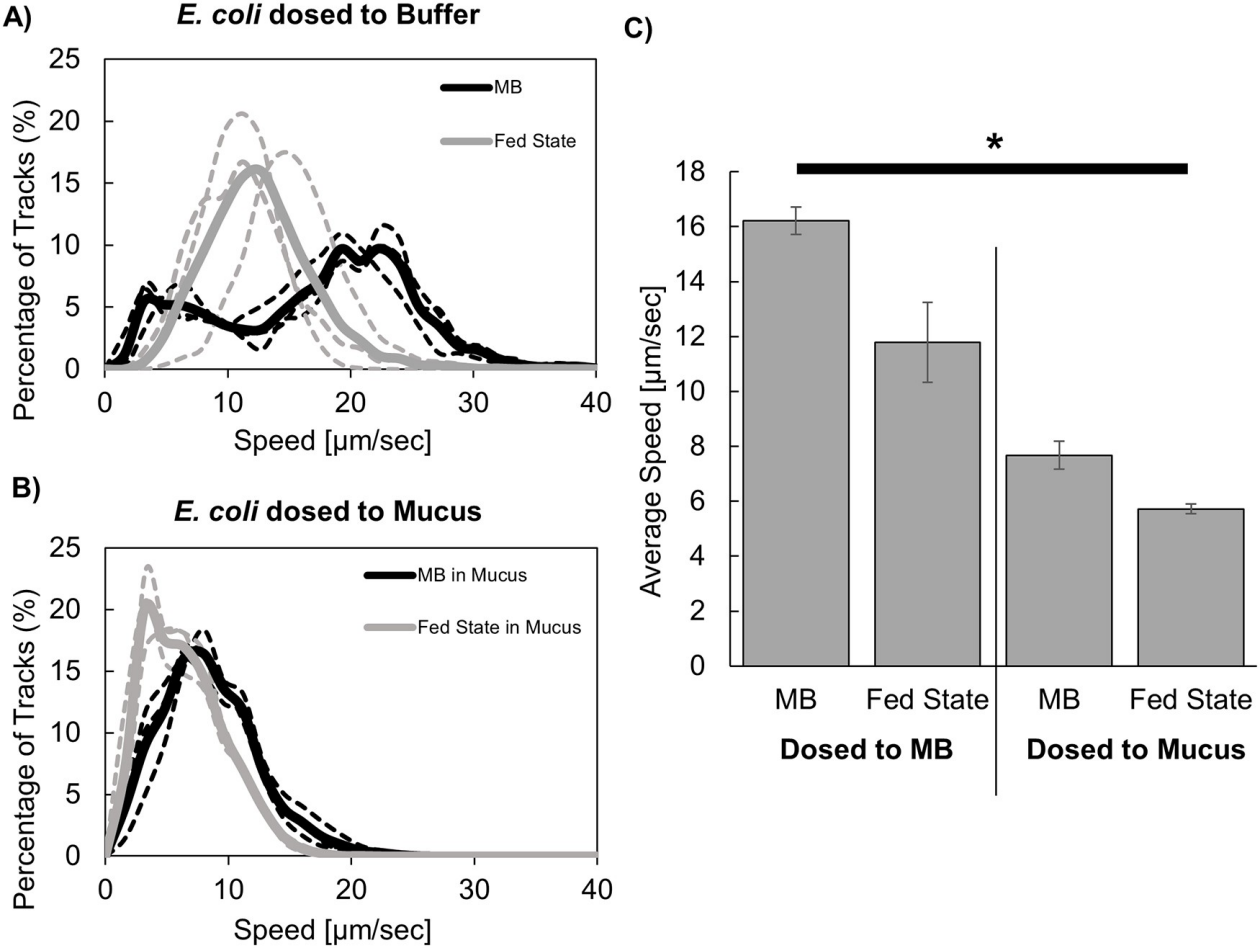
*E*. *coli* speed in buffer and mucus after dosing to buffer or fed state medium. Distribution of *E*. *coli* speeds in A) MB or B) mucus, after dosing in MB (black) or fed state medium (grey). Dashed lines represent individual experiments, and solid lines represent the overall average. C) Average speed of *E*. *coli* in MB or mucus after dosing in MB or fed state medium. Data represent mean ± standard error of three independent experiments, with *n* ≥100 microbes for each experiment, and significance was observed between all groups (*p < 0.05).

Visual inspection of microbe trajectories (Fig 2A and 2B, S1 and S2 Videos) and quantitative analysis of track linearity (Fig 2E) indicated fed state medium had minimal impact on microbe swimming patterns within MB. Microbial speeds in MB spanned similar ranges in the absence and presence of fed state medium: 42.97 to 0.83 μm/sec and 41.54 to 1.72 μm/s, respectively (Fig 3A). In the absence of fed state medium, *E*. *coli* speed in MB presented a bimodal distribution, which can reflect a fraction of the population being highly motile or non-motile [18, 19]. Interestingly, the presence of fed state medium resulted in a unimodal *E*. *coli* speed distribution and a significantly decreased average microbe speed in MB (11.79 μm/sec relative to 16.21 μm/sec in the absence of fed state medium, Fig 3A). The fed state medium is a colloid-rich solution consisting of model bile micelles (reported to have dimensions of 5.9–12.7 nm (length) by 3.6–4.1 nm (width) [20]) and triglyceride microemulsion droplets (avg. diameter 1147.2 ± 72.5 nm). The microemulsion droplets are stabilized by amphiphilic molecules (bile salt and phospholipid, as well as triglyceride digestion products sodium oleate and 1-oeoyl-rac-glycerol) coating their surfaces and separating water- and oil- rich domains [21, 22]. These lipid microemulsion droplets may act as impenetrable obstacles to the movement of *E*. *coli*, thus contributing to slower microbial speeds. The presence of model bile (0.9% wt/vol.) and lipids (3.05% wt/vol.) also significantly increased the viscosity of MB (0.86 ± 0.016 vs. 1.11 ± 0.019 cSt), which may have contributed to the decreased motility and altered track linearity (Fig 2E) due to increased hydrodynamic drag experienced by the *E*. *coli*.

**Fig 4:**
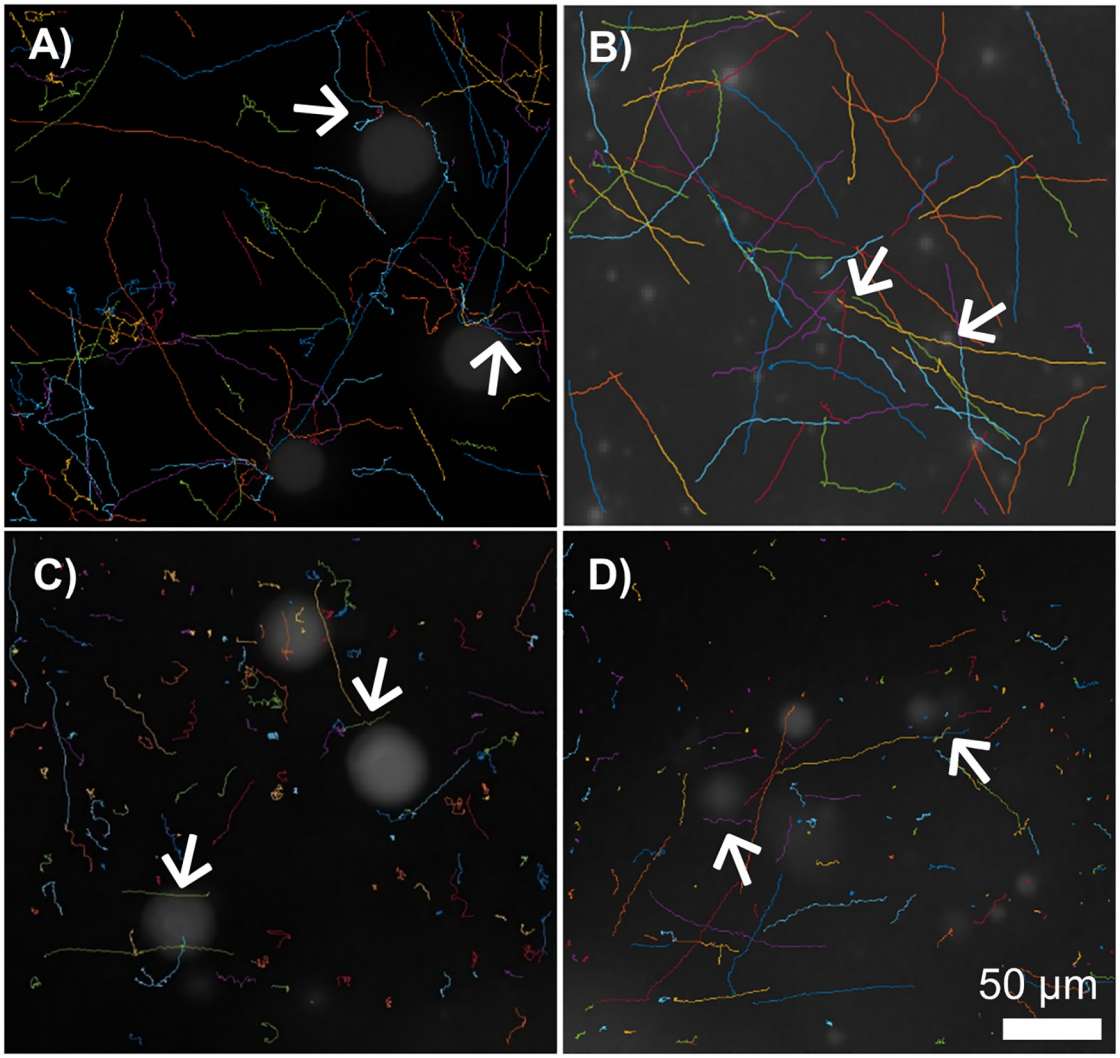
Representative 20 s trajectories of *E*. *coli* in Fed State dosed to A-B) MB or C-D) mucus. Lipid emulsions were stained with BODIPY to enable visualization of microbe associations with emulsions (20x magnification). A&B and C&D are two representative pictures of the conditions in MB and in mucus, respectively. Bacteria that appeared to come in contact with emulsion droplets associated with droplet surfaces for some time before continuing on in bulk solution (→). Scale bar = 50 μm.

Investigation via video-microscopy of microbe trajectories relative to droplet interfaces revealed that direct interactions between *E*. *coli* and lipid microemulsion droplets in fed state medium visibly altered microbial movement. When *E*. *coli* came in contact with emulsion droplets, they appeared to associate with the surface such that their trajectories followed the microemulsion droplet surface for some time before continuing on through the bulk medium (S3 and S4 Videos, Fig 4A and 4B). Complex interfaces, including surfactant-covered interfaces, can re-direct flagellated microbial motion such that swimming paths align parallel to the interfacial surface [23].

### Impact of fed state medium on the transport of *E*. *coli* within mucus

*E*. *coli* swimming patterns within mucus were markedly altered relative to those within MB, with a significant increase in the percentage of rotating tracks and a decrease in microbial speed; these differences may be attributed to the complex mucus structure and potential binding interactions with mucus components (Figs 2 and 3). *In vivo*, microorganisms must penetrate the protective mucus layer to adhere to or invade the gastrointestinal epithelium. Within mucus, mucin glycoprotein oligosaccharides can act as ligands for bacteria and viruses, in some cases blocking the binding of microorganisms to the intestinal epithelium and thus protecting against gastrointestinal infection[24]. It is interesting to note that a unimodal speed distribution was observed within mucus, as opposed to the bimodal distribution in MB. This could suggest that in MB alone, relatively non-motile bacteria may be somewhat stuck to glassware, and that the presence of a complex matrix such as mucus or the presence of lipids prevents the interactions with glass.

**Fig 5:**
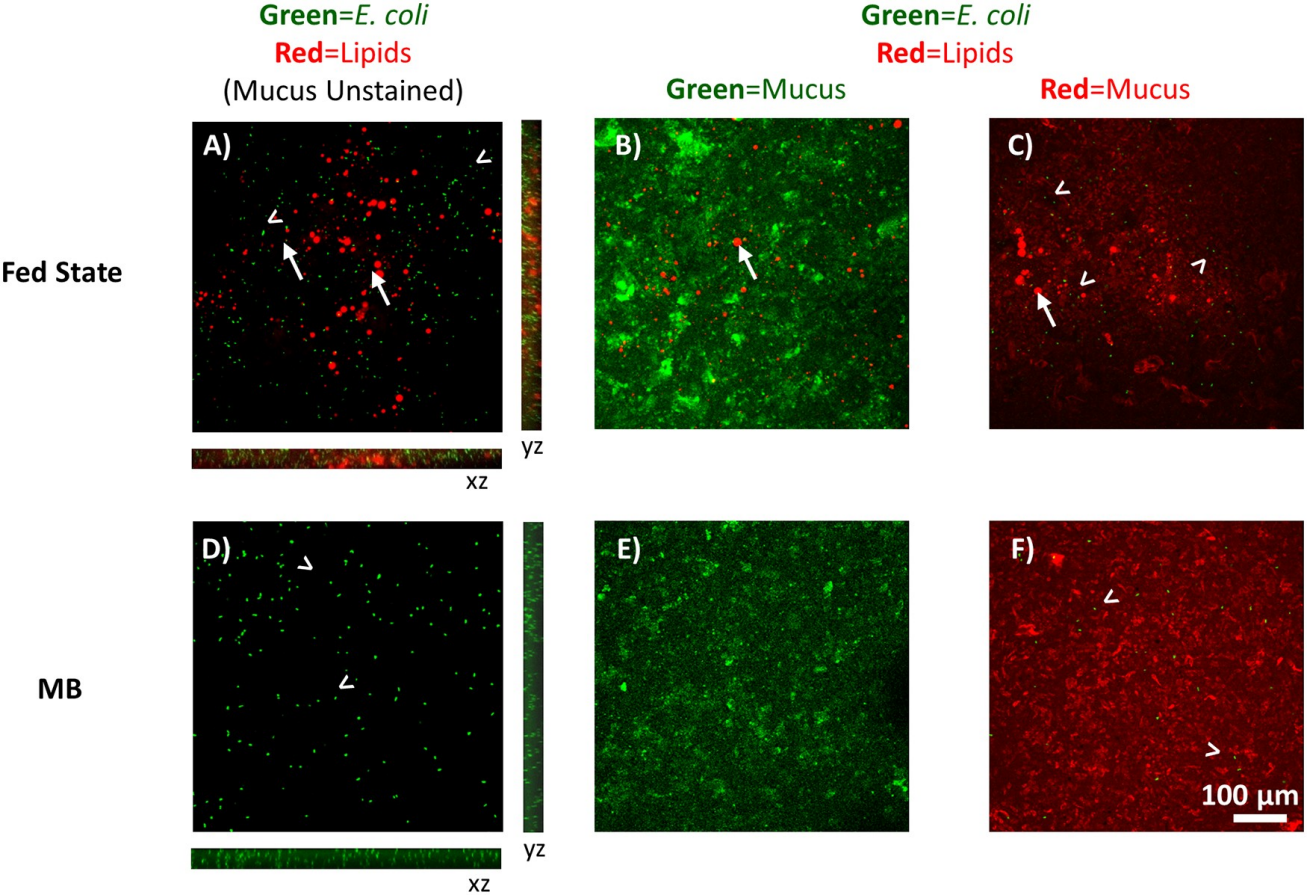
Lipid and *E*. *coli* distribution in mucus. Z-projection of *E*. *coli* (green) distribution in mucus (unstained) after exposure to A) fed state medium containing lipid microemulsions (red, →) or D) MB. Orthogonal cross-sections are 47 μm thick. Distribution of lipid microemulsion (red, →) in mucus (green) after exposure to B) fed state medium or E) MB. Distribution of *E*. *coli* (green) and lipid microemulsion (red, →) in mucus (red) after exposure to C) fed state medium or F) MB. Scale bar = 100 μm.

The presence of fed state medium significantly impacted *E*. *coli* mobility in mucus, decreasing average speed from 7.76 μm/sec in the absence of exogenous lipids (range of 26.56 to 0.49 μm/sec), to 5.63 μm/sec (range of 22.795 to 0.33 μm/sec) (Fig 3). Interestingly, although the presence of fed state medium decreased average speed to a similar extent (~1.3-fold) in MB and mucus, analysis of the microbe trajectories indicates the mechanism by which lipids impact speed in mucus differs from that in buffer. Fed state medium decreased the relative number of curvilinear tracks and increased the relative number of rotating tracks in mucus (Fig 2C–2E). Lipid microemulsions visibly appeared to maintain structural integrity upon introduction of fed state medium to mucus (Fig 5A and 5B). While surface association of *E*. *coli* with microemulsion surfaces was evident in analysis of trajectories in both MB and mucus, it was observed less frequently within mucus, potentially due to hindered mobility within mucus preventing microbes from traveling significant distances during the short timeframe of collected videos (Fig 4C and 4D, S5 and S6 Videos). Since microemulsions visibly remained intact within mucus, they may act as crowding agents, thereby decreasing the available pore volume and altering microbe mobility and swimming trajectories. In addition, the impact of fed state medium on swimming patterns and speed of microbes dosed in mucus is likely due in part to changes in the mucus network, and associated impact of altered mucus structure on microbe motility. Microemulsions (composed of medium chain triglyceride, ethyl oleate, castor oil, Cremophor RH40, and 1,2-propanediol) were previously found to interact with and closely adhere to mucin fibers, increasing viscosity in a 2% w/w mucin solution[25]. Moreover, microrheological analysis demonstrated the addition of fed state to mucus altered mucus viscoelasticity, increasing the elastic modulus to a greater degree than the viscous modulus, and significantly decreasing particle diffusion and confining particles to defined regions within the mucus gel[10]. This was attributed to changes in mucus microstructure resulting from non-covalent associations between lipids and mucus, potentially including hydrophobic interactions with non-glycosylated portions of the mucin backbone or H-bonding with glycosylated portions[26–28]. Interestingly, exposure of mucus to FS medium did not appear to result in confinement of *E*. *coli* to specific areas as had been observed with particles. Rather *E*. *coli* distributions within mucus in the presence and absence of lipids were similar (Fig 5C and 5F). Thus, the decreased transport of microbes through mucus may occur mainly due to surface association or crowding by lipid microemulsions.

The finding that lipids impact microbe mobility *in vitro* is of significant interest with respect to potentially impacting microbe mobility *in vivo*. However, co-dosing microbes with test media could introduce confounding effects of harsh acidic gastric contents on microbe mobility. Nonetheless, previous observation that oral dosing of lipids impacted mucosal penetration of particles[10] supports the potential translation of the decrease in microbial transport *in vitro* to *in vivo*. Yildiz et al. showed particles penetrated significantly further into mucosa from animals fed water compared to animals fed lipid (soybean oil).

**Fig 6:**
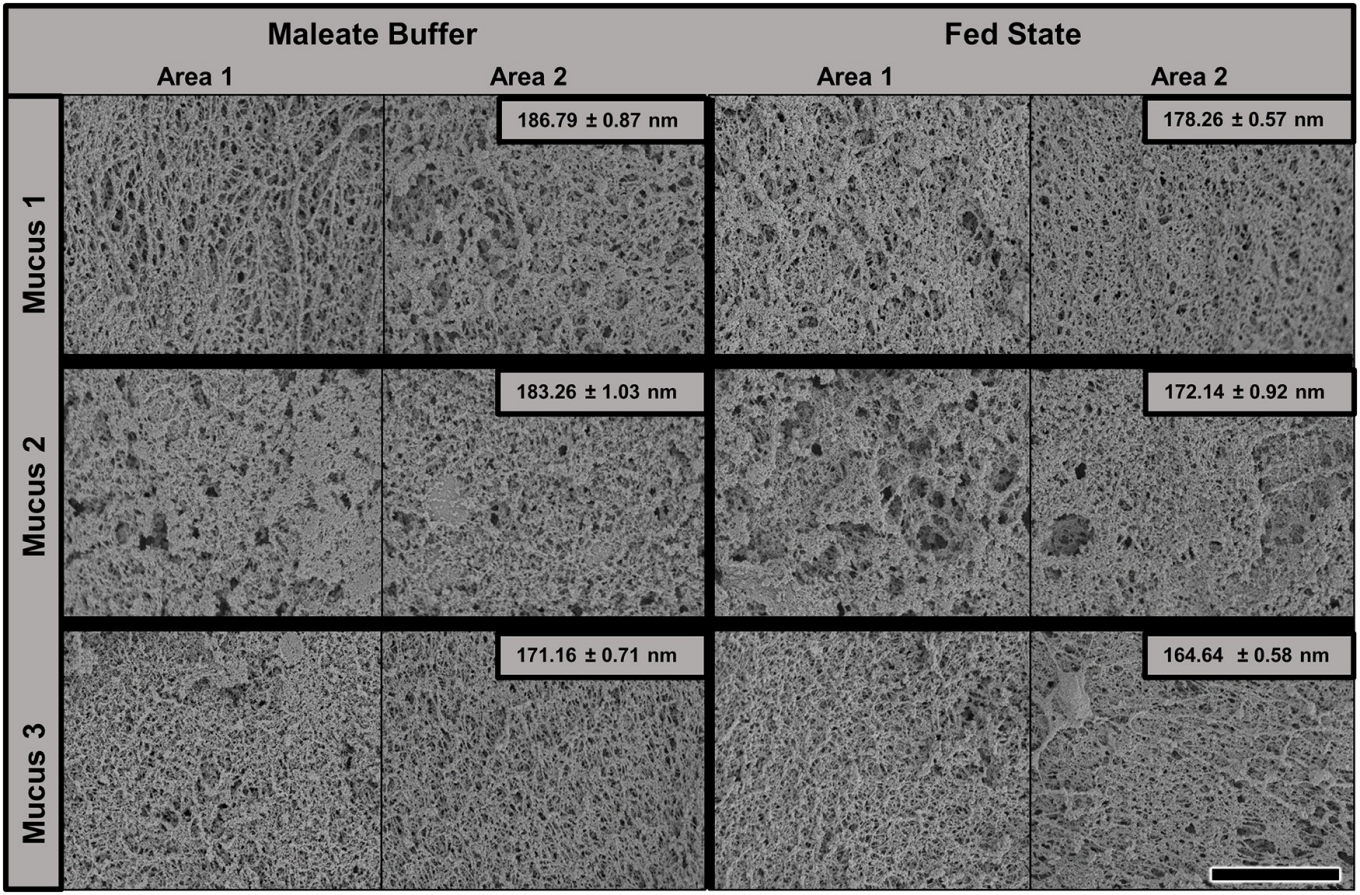
SEM micrographs of porcine mucus after exposure to MB or fed state medium. Mucus was collected from three different animals (Mucus 1, 2, 3). Individual sample pore sizes are shown. Scale bar = 5μm.

To investigate potential impact of fed state medium on mucus ultrastructure, scanning electron microscopy was utilized. Mucus microstructure as evident by SEM was not visibly impacted by fed state medium. However, the presence of fed state medium did result in a subtle decrease in average pore size (172.5 ± 0.38 nm vs 180 ± 0.49 nm) (Fig 6).

## Conclusion

In this study, we have shown that lipids present in fed state medium simulating intestinal lumen contents after eating can significantly impact microbe transport, reducing speed and altering swimming patterns. The lipid microemulsion droplets present in fed state medium visibly maintained integrity upon introduction to buffer and mucus and acted as obstacles altering *E*. *coli* swimming patterns, as microbes were observed to associate with microemulsion surfaces for a period of time before continuing to swim through bulk medium. Furthermore, lipids altered the linearity of *E*. *coli* trajectories within mucus, potentially in part by acting as crowding agents, decreasing the effective pore volume available for transport. As lipids are normal food components and thus represent safe, mild stimuli, these results support exploration of lipid-based strategies to alter the mucus barrier to control microbe invasion of epithelium underlying mucosal surfaces.

## Supporting information

S1 Video Representative 20 s movie, with trajectories, of *E*. *coli* swimming in MB (40x magnification). (AVI)

S2 Video Representative 20 s movie, with trajectories, of *E*. *coli* exposed to fed state medium swimming in MB (40x magnification). (AVI)

S3 Video Representative 20 s movie of *E*. *coli* exposed to fed state medium swimming in MB. Lipid emulsions (red) were stained with BODIPY to enable visualization of microbe associations with emulsions (20x magnification).
(AVI)

S4 Video Representative 20 s movie, with trajectories, of *E*. *coli* exposed to fed state medium swimming in MB. Lipid emulsions (white) were stained with BODIPY to enable visualization of microbe associations with emulsions (20x magnification).
(AVI)

S5 Video Representative 20 s movie of *E*. *coli* exposed to fed state medium swimming in mucus. Lipid emulsions (red) were stained with BODIPY to enable visualization of microbe associations with emulsions (40x magnification).
(AVI)

S6 Video Representative 20 s movie, with trajectories, of *E*. *coli* exposed to fed state medium swimming in mucus. Lipid emulsions (white) were stained with BODIPY to enable visualization of microbe associations with emulsions when dosed to mucus. (40x magnification).
(AVI)
